# Functional development of olfactory tubercle domains during weaning period in mice

**DOI:** 10.1038/s41598-018-31604-1

**Published:** 2018-09-04

**Authors:** Wataru Murofushi, Kensaku Mori, Koshi Murata, Masahiro Yamaguchi

**Affiliations:** 1 Department of Physiology, Graduate School of Medicine, the University of Tokyo, Tokyo, 113-0033 Japan; 2 Division of Brain Structure and Function, Faculty of Medical Sciences, University of Fukui, Fukui, 910-1193 Japan; 3 Life Science Innovation Center, Faculty of Medical Science, University of Fukui, Fukui, 910-1193 Japan; 4 Department of Physiology, Kochi Medical School, Kochi University, Kochi, 783-8505 Japan

## Abstract

Mammals shift their feeding habits from mother’s milk to environmental foods postnatally. While this weaning process accompanies the acquisition of attractive behaviour toward environmental foods, the underlying neural mechanism for the acquisition is poorly understood. We previously found that adult mouse olfactory tubercle (OT), which belongs to the olfactory cortex and ventral striatum, has functional domains that represent odour-induced motivated behaviours, and that c-fos induction occurs mainly in the anteromedial domain of OT following learned odour-induced food seeking behaviour. To address the question whether the anteromedial OT domain is involved in the postnatal acquisition of food seeking behaviour, we examined OT development during weaning of mice. Whereas at postnatal day 15 (P15), all mice were attracted to lactating mothers, P21 mice were more attracted to familiar food pellets. Mapping of c-fos induction during food seeking and eating behaviours showed that while c-fos activation was observed across wide OT domains at P15, the preferential activation of c-fos in the anteromedial domain occurred at P21 and later ages. These results indicate that preferential c-fos activation in the anteromedial OT domain occurred concomitantly with the acquisition of attractive behaviour toward food, which suggests the importance of this domain in the weaning process.

## Introduction

Shortly after birth, mammals begin to suckle their mother’s nipple and drink breast milk, whose nutrition is adapted for the optimal growth of early neonates. Then they begin to eat foods of environmental origin and become completely dependent on environmental food for nutrition. This shift in feeding habits, called weaning, is a fundamental growth process of postnatal mammals during which they shift from mother-dependent to mother-independent sustainment of life^1–3^. The time course of weaning has long been documented in rats. Rat pups begin to consume environmental food such as food pellets typically around the third week after birth, during which suckling of the mother’s nipple and consumption of environmental food co-exist. Thereafter, food eating gradually replaces milk suckling and rats become independent from their mother within 1 month of birth^4–7^. Weaning is considered to be a learning process. The identification of edible objects mostly depends on experience, and food experience at a younger age evokes personal food preferences, which are generally maintained throughout life^8^. Despite the importance of weaning for animals’ food-eating life, the underlying neural mechanisms for the shift in feeding habits are not well understood.

Food-eating behaviour is highly dependent on chemical senses. Animals search for food via odour cues, and judge whether to accept or reject objects by integrating odour, taste and oral tactile sensations^9^, which indicates the crucial role of olfaction in the acquisition of food-eating behaviour. In other words, olfaction is tightly coupled with the induction of motivated behaviours such as food seeking and food consumption. Preferred food odour from the external environment elicits attractive motivated behaviours^10^. Thus, the knowledge of neural circuit mechanism that translates food odour information to eating-related attractive motivated behaviours seems to be the key to understanding the acquisition of food-eating behaviour during weaning process.

Recent advances in studies of the olfactory cortex have highlighted the olfactory tubercle (OT) as a candidate structure that links odour cues to motivated behaviours^11–13^. Because the OT receives direct inputs from the olfactory bulb, the first relay centre of olfactory information processing, it is a part of the olfactory cortex^14^. In addition, the OT contains medium spiny neurons as principal neurons that receive massive dopaminergic inputs from the midbrain, and constitutes a ventral striatum with the nucleus accumbens^11–13,15–17^. In line with these structural properties, functional studies have supported the notion that the OT is involved in the translation of sensory signals into various motivated behaviours^18–23^.

We previously showed that the OT has domain structures that represent odour-induced motivated behaviours in mice. By associating an odour with a food reward or electrical foot shock punishment, mice learn to exhibit attractive or aversive behaviours, respectively, to the learned odour. Importantly, c-fos activation occurs preferentially in the anteromedial domain of the OT during learned odour-induced food seeking behaviour, whereas preferential c-fos activation occurs in the lateral domain during learned odour-induced aversive behaviour^24^. This observation raises the question of whether the OT, particularly the anteromedial domain, is involved in the postnatal acquisition of food-eating behaviour during the weaning process.

In contrast to observations in rats, detailed analyses of the weaning process in mice have been limited. While rats begin to consume food pellets at approximately P17 and cease to consume mother’s milk at approximately P27^4–7^, timing of these shifts in feeding behaviour is not well addressed in mice. Here, we examined the weaning process of mice in detail and compared its time course with that of OT domain development. The results indicate that the structural and functional development of the anteromedial OT domain occurs concomitantly with the shift of feeding habits during weaning process.

## Results

### Shift in feeding behaviour during the postnatal period

We first examined the time course of feeding habit shift of postnatal C57BL/6 mice from mother’s milk to environmental food. Mice at different postnatal ages were individually housed in a new cage for 6–16 h depending on the age (see Methods), without their mothers or food pellets, and assigned to two groups. One group of mice was presented with their mother under anaesthesia, and feeding behaviour was evaluated by attachment to the mother’s nipple. The second group of mice was presented with food pellets, and feeding behaviour was evaluated by the consumption of food pellets. For both groups, the behaviours of P8, P15, P21, P27, and P56 mice (n = 10 for each condition) were examined for 30 min following delivery of lactating mothers or food pellets.

**Figure 1:**
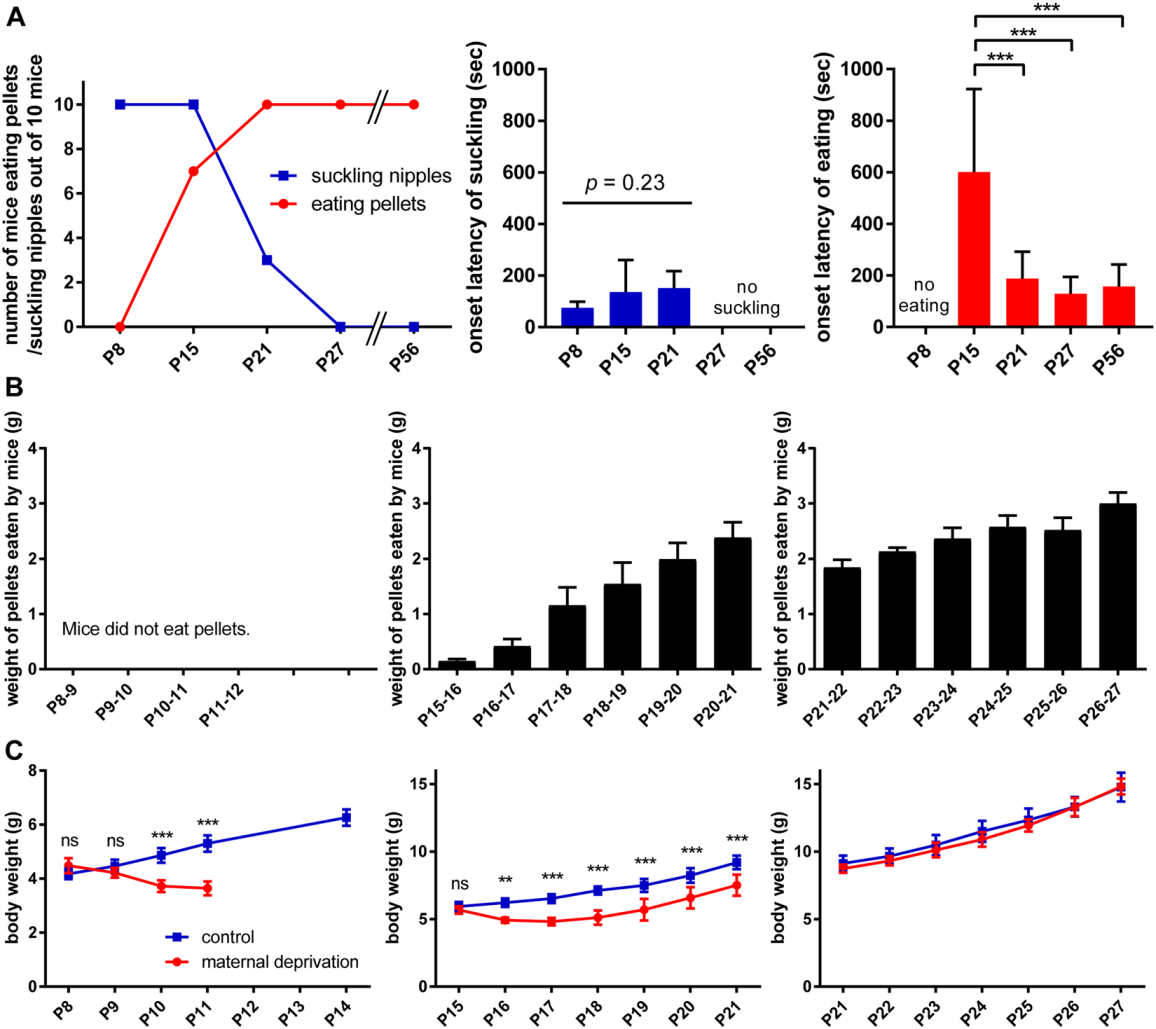
Access to and growth dependence on feeding objects in postnatal mice during the weaning period. (**A**) Feeding behaviour following delivery of lactating mothers or food pellets. An anaesthetised mother or food pellet was presented and behaviour was observed for 30 min. Left panel, number of mice that suckled nipples or consumed food pellets examined at different ages (n = 10). Middle and right panels, latency to the first approach to feeding objects. Latency to lactating mothers (middle) and food pellets (right) are shown. Data represents the mean ± standard deviation (SD). ^***^p < 0.001 (one-way ANOVA with *post hoc* Tukey’s test). (**B**) Volume of food pellets consumed each day, starting at postnatal day 8 (P8; left), P15 (middle), and P21 (right) in individual housing conditions. Data represents the mean ± SD (n = 5). (**C**) Comparison of body weight under maternal deprivation and a control condition housed with the mother. Data represents the mean ± SD (n = 5). ^**^p < 0.01; ^***^p < 0.001; ns, not significant (n = 5, two-way ANOVA with *post hoc* Tukey’s test).

All P8 and P15 mice approached the mother’s nipple and attached within 30 min of delivery of the lactating mother (Fig. 1A, left panel, blue line). Active suckling with the appearance of milk in the stomach were confirmed in all mice by dissecting the stomach following the behavioural analysis and sacrifice under deep anaesthesia (data not shown). By contrast, only three mice at P21 and no mice at P27 or P56 attached to the mother’s nipple. In the case of food pellet delivery (Fig. 1A, left panel, red line), P8 mice did not exhibit pellet-eating behaviour. Seven P15 mice consumed food pellets within the test period, and all mice at P21, P27, and P56 consumed food pellets. All mice at P8 and three P15 mice, which showed no pellet-eating behaviour, did not even approach the food pellet.

Latency to the first approach to the mother’s nipple or food pellets during the 30 min of delivery was also examined. Latency to the nipple approach of mice at P8, P15 and P21 mice was mostly less than 3 min, which was not statistically different among different ages (Fig. 1A, middle panel). Latency to the food pellet approach of mice at P15 was about 10 min on average and that at P21, P27 and P56 was about 3 min on average, where the latency of P15 mice was significantly longer than that of older mice (Fig. 1A, right panel).

These observations indicated that P8 mice exhibited only milk-consuming behaviour, P15 and P21 mice showed both milk- and food pellet-consuming behaviours, and P27 and P56 mice exhibited only food pellet-consuming behaviour. This time course of shift in feeding behaviour is similar to previous observations in rats^3–7^. In addition, the longer latency to food pellet approach of P15 mice raised the possibility that food-pellet eating behaviour was under development at this period.

Next, we addressed the question of to what extent mice in different postnatal ages depended on environmental food for nutrition, by observing their feeding behaviour and growth over days. P8, P15, or P21 mice were individually transferred to new test cages followed by delivery of food pellets. The mice were housed in the test cage for several days, and the amount of food pellets consumed (Fig. 1B) and body weight (Fig. 1C) were measured daily. P8 mice did not consume food pellets until at least P12 (Fig. 1B, left panel). During this period, their body weight declined to an average of approximately 70% of the initial body weight (Fig. 1C, left panel). P15 mice consumed a small amount of food pellets on the first day, and the amount consumed increased progressively (Fig. 1B, middle panel). The body weight of P15 mice declined during the first 3 days and then began to increase, but did not catch up with control mice until at least P21 (Fig. 1C, middle panel). In contrast, P21 mice consumed a significant amount of food pellets on the first day and thereafter, and their body weight increased in a similar manner as control mice (Fig. 1B,C, right panels). These observations indicated that food pellets were not a source of nutrition at all for P8-P12 mice, and that food pellets were not a major source of nutrition even for P15 mice while they began to develop food-eating behaviour. On the other hand, P21 mice showed significant food-eating behaviour and obtained sufficient nutrition from food pellets.

**Figure 2:**
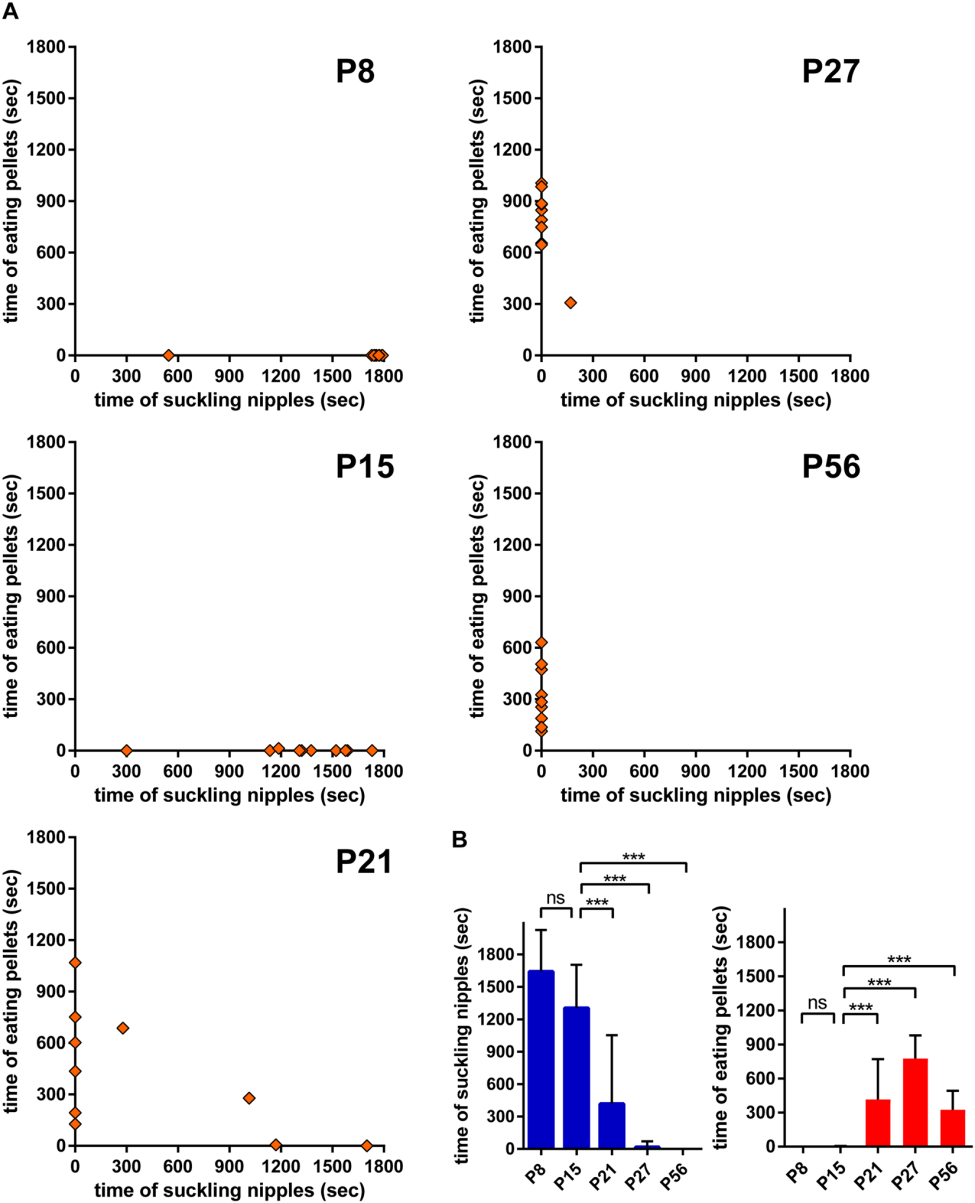
Feeding shift from mother’s milk to food pellets between P15 and P21. (**A**) Time spent suckling mother’s nipple and eating pellets during feeding object presentation (30 min). An anaesthetised mother and food pellet were simultaneously presented. Each plot represents the data of one mouse (n = 10). (**B**) Statistical comparison of the time spent nipple suckling (left) and pellet eating (right). Data represents the mean ± SD. ^***^p < 0.001; ns, not significant (n = 10, one-way ANOVA with *post hoc* Tukey’s test).

Then we examined which object, lactating mothers or food pellets, is the major attractant for postnatal mice by simultaneous presentation of the two objects. Mice at different postnatal ages were individually housed in a new cage for 6–16 h without mothers or food pellets and presented with the lactating mother and food pellets simultaneously (Fig. 2). The length of time of attachment to the mother’s nipple or pellet eating was measured during the 30 min test period (n = 10 for each condition). All P8 and P15 mice attached to the mother’s nipple during most of the test period, whereas they showed almost no pellet-eating behaviour (Fig. 2A). By contrast, significant pellet-eating behaviour was observed for P21 mice. Among the 10 mice, 6 exhibited pellet-eating behaviour but no attachment to the mother’s nipple, and 2 exhibited both pellet-eating behaviour and nipple attachment. The remaining two mice were exclusively attached to the mother’s nipple. In P27 mice, pellet-eating behaviour further dominated, similar to P56 adult mice.

Quantification of the time length of feeding behaviour (Fig. 2B) showed that attachment to the mother’s nipple was evident at P8 and P15, declined at P21, and was nearly absent at P27 (Fig. 2B, left). By contrast, pellet-eating behaviour was not evident at P8 and P15, and only observed at P21 and later (Fig. 2B, right). Thus, the simultaneous presentation of the two feeding objects indicated that P8 mice were exclusively attracted to the mother’s nipple, P15 mice were more attracted to the mother’s nipple, P21 mice were more attracted to food pellets, and P27 and P56 mice were almost exclusively attracted to food pellets.

Collectively, these observations demonstrated the time course of the postnatal shift in feeding behaviour. P8 mice depended exclusively on the mother’s milk for nutrition and had not yet developed food-eating behaviour. P15 mice began to show food-eating behaviour, although they mostly depended on the mother’s milk for nutrition and were more attracted to lactating mothers than food pellets. P21 mice showed significant food-eating behaviour, obtained sufficient nutrition from food pellets, and were more attracted to food pellets than lactating mothers. P27 and P56 mice were exclusively attracted to food pellets and depended solely on food pellets for nutrition.

### Structural development of the OT during the postnatal period

Then we examined the structural development of the OT during the postnatal period. In adult mice, the OT has a three-layered cortex-like structure where medium spiny neurons are aligned to constitute a middle layer. The adult OT is divided into several compartments by cell-dense structures called cap compartments and the Islands of Calleja^12,16,24–26^. The cap compartments consist of densely packed small-sized medium spiny neurons and are distributed at the superficial portion of the lateral OT^16,26^. The islands of Calleja, which contain densely packed granule cells and local interneurons, are distributed across wide areas of the OT^12,24,25^. Using these cell-dense structures as landmarks, we previously defined four domains of the cortex-like structures in the adult OT; i.e., anteromedial, lateral, central, and posterior domains^24^. The cap compartments and the Islands of Calleja can be discriminated by location as well as by molecular expression. Medium spiny neurons in the cap compartments express DARPP-32, a signalling molecule downstream of dopamine receptors^27^, whereas granule cells in the Islands of Calleja are negative for DARPP-32^24^.

**Figure 3:**
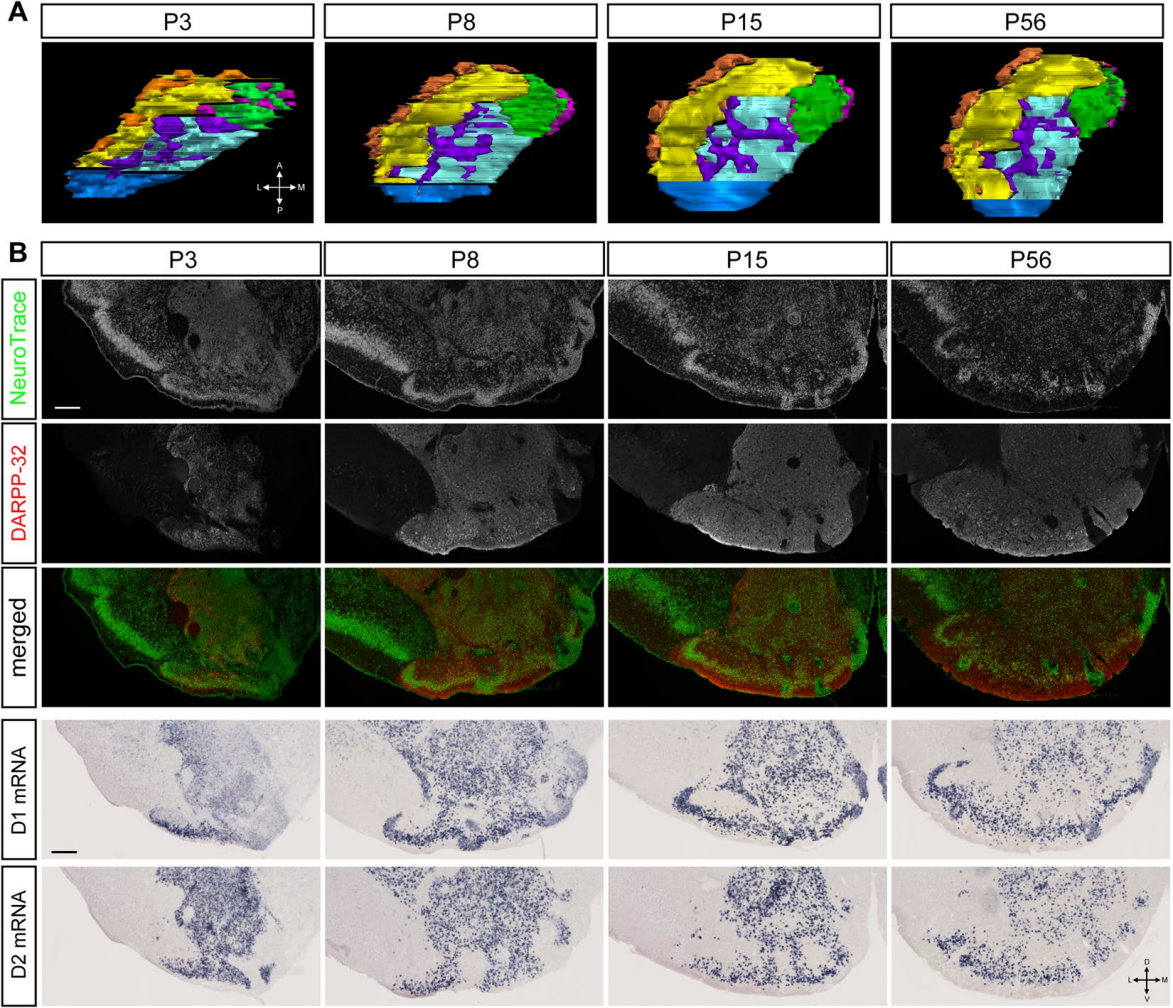
Domain structures of the postnatal OT and postnatal maturation of the anteromedial OT domain. (**A**) Reconstruction of the cap compartment, Islands of Calleja, and cortex-like domain of the OT at P3, P8, P15, and P56 in the left hemisphere (dorsal view). Orange, cap compartment; magenta, superficial Islands of Calleja; purple, deep Islands of Calleja; green, anteromedial cortex-like domain; yellow, lateral cortex-like domain; cyan, central cortex-like domain; blue, posterior cortex-like domain. (**B**) Molecular expression in the OT domains. The panels are coronal sections of the OT at postnatal periods indicated. Top row, NeuroTrace staining; second row from top, immunostaining for DARPP-32; middle row, merged view of NeuroTrace (green) and DARPP-32 (red). Second row from bottom, *in situ* hybridisation for dopamine receptor type 1 (D1) mRNA; bottom row, dopamine receptor type 2 (D2) mRNA. Scale bars: 200 μm. A, anterior; P, posterior; L, lateral; M, medial; D, dorsal; V, ventral.

Coronal sections of the OT at various postnatal periods were prepared and examined for the compartmentalised structure (Fig. 3). Fluorescent Nissl staining indicated that the OT of P3 mice showed a three-layered structure with cell-dense structures, similar to the adult OT. DARPP-32 immunoreactivity was already evident in the OT of P3 mice, which enabled identification of the cap compartments and Islands of Calleja and revealed the compartmentalised structure including the four cortex-like domains, the lateral cap compartment, and the superficial and deep Islands of Calleja as shown in a two-dimensional flattened map (Fig. 3A). OT compartments were also evident in P8 and P15 mice (Fig. 3A). The overall compartmentalised OT structure during these postnatal periods was similar to that of adult mice at P56, which suggests that the compartmentalised structure of the OT developed early and was present before the start of weaning.

**Figure 4:**
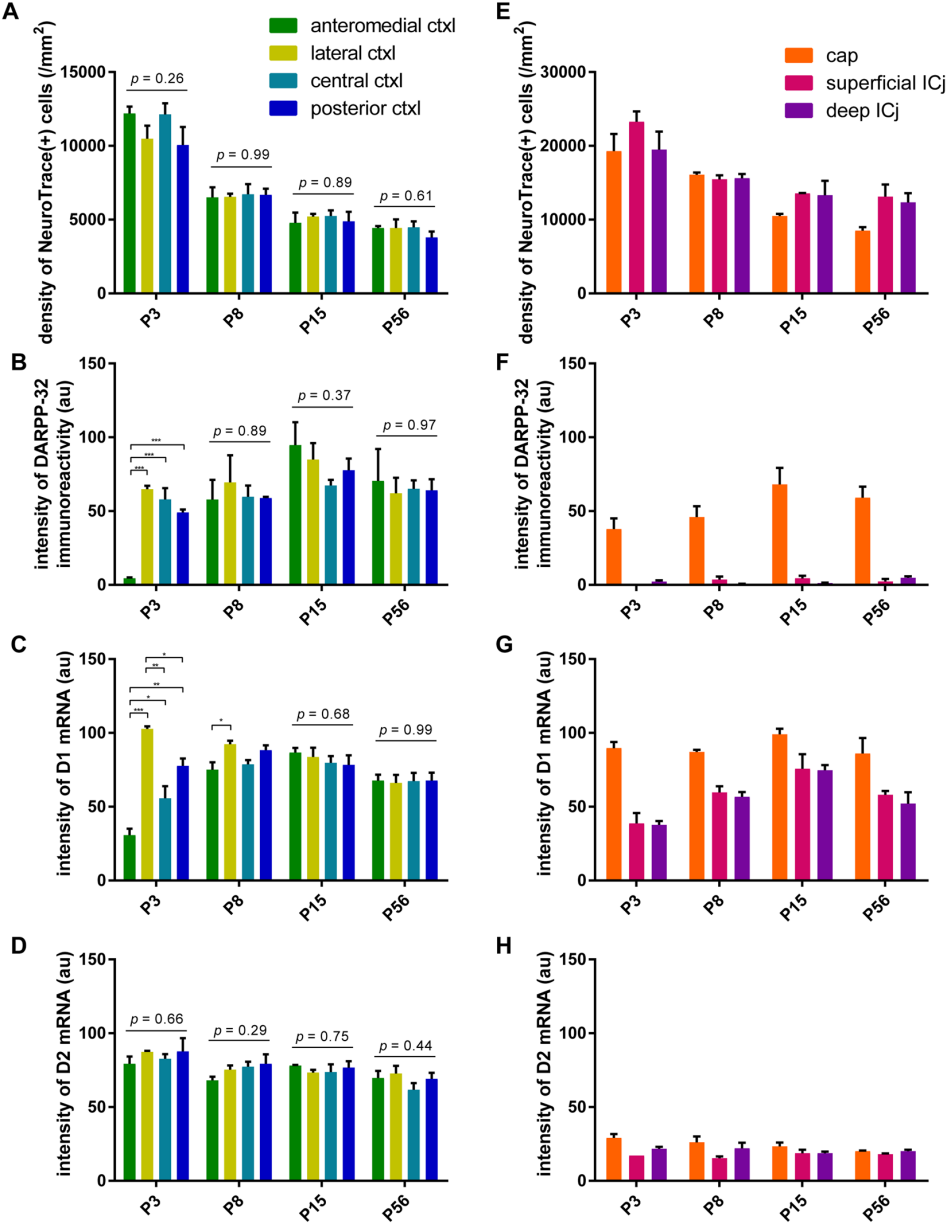
Quantification of the postnatal development of the OT compartments. The OT was divided into four cortex-like domains, cap compartment, superficial Islands of Calleja (ICj), and deep ICj. In each compartment, the density of NeuroTrace (+) cells (**A**,**E**), intensity of DARPP-32 immunoreactivity (**B**,**F**), intensity of D1 mRNA signal (**C**,**G**), and intensity of D2 mRNA signal (**D**,**H**) are shown. (**A**–**D**) Quantification in cortex-like domains. (**E**–**H**) Quantification in cap compartment and ICj. Data represent the mean ± SEM. Statistical significance was calculated using one-way ANOVA with *post hoc* Tukey’s test to compare signal intensity among the four cortex-like domains (n = 3). ^*^p < 0.05; ^**^p < 0.01; ^***^p < 0.001.

In contrast to the early structural development, analyses of molecular expression revealed early neonatal immaturity and postnatal maturation of OT compartments. DARPP-32 immunoreactivity was present but weak in P3 mice, particularly in the medial part of the OT (Fig. 3B). Compartment-based quantification showed that, while cell density was equivalent among the different cortex-like domains from as early as P3 (Fig. 4A), the intensity of DARPP-32 immunoreactivity was remarkably weaker in the anteromedial cortex-like domain at P3 (Fig. 4B) compared with other three domains. The DARPP-32 immunoreactivity in the anteromedial domain increased to the level comparable to the other three domains at P8 and later. Furthermore, dopamine receptor type 1 (D1) mRNA expression was significantly weaker in the anteromedial cortex-like domain at P3, and this weaker expression in the anteromedial domain continued until P8 (Figs 3B and 4C). On the other hand, dopamine receptor type 2 (D2) mRNA expression in the anteromedial domain was not significantly different from other cortex-like domains throughout the observed period (Figs 3B and 4D).

These observations imply that functional maturation of the OT occurs during the postnatal weaning period. In particular, development of the anteromedial cortex-like domain might be delayed at P3 and P8 compared to the remaining 3 domains. On the other hand, cap compartments and the Islands of Calleja appeared to have been well developed at birth, based on cell density and molecular expression (Fig. 4E–H).

### Activation of OT domains during food-eating behaviour in postnatal mice

We previously showed in adult mice that increase in c-fos mRNA expression occurs preferentially in the anteromedial OT domain when mice showed odour-induced food-expecting, attractive behaviour^24^. Therefore, we examined the c-fos activation in individual domain of the OT in postnatal mice during food-eating behaviour. Mice at different postnatal ages were individually housed in a new cage for 6–16 h without their mothers or food pellets, followed by delivery of food pellets. After 30 min of observation, the mice were perfusion-fixed and c-fos mRNA expression in individual domain was analysed. Mice not presented with food pellets were analysed as controls.

**Figure 5:**
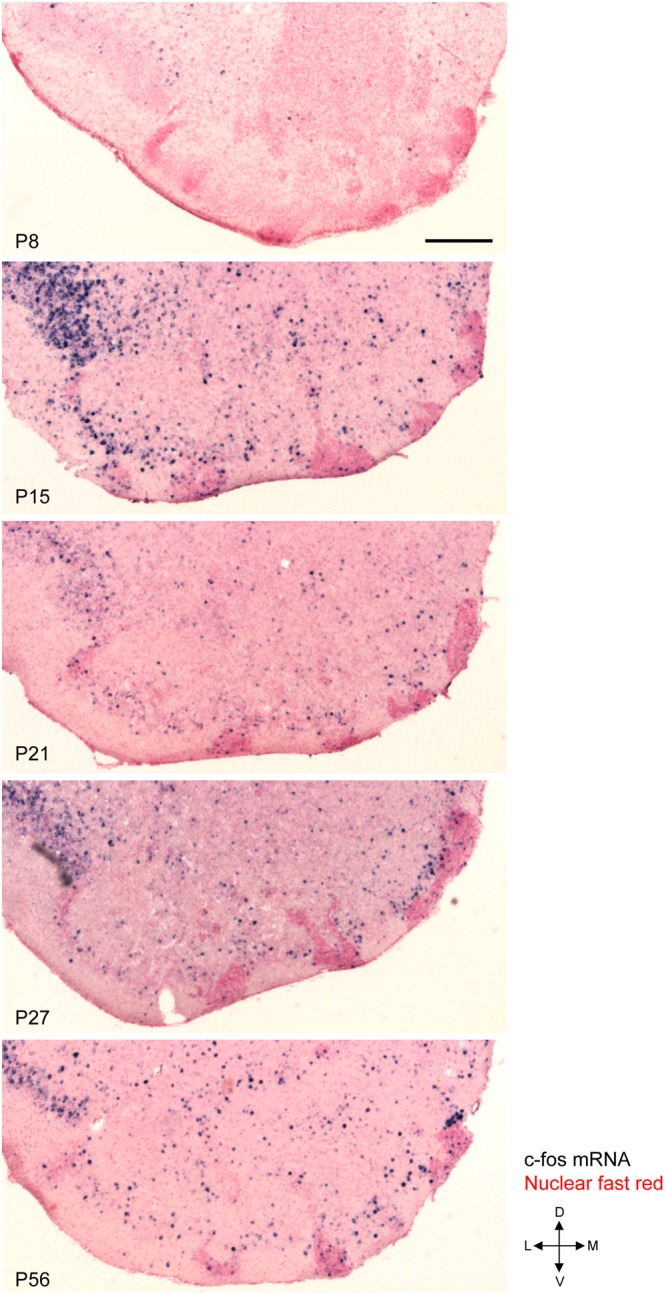
c-fos activation in the OT following access and consumption of food pellets in postnatal mice. Mice at various postnatal periods were presented with food pellets for 30 min and activation of the OT was examined by c-fos mRNA expression. The panels show the distribution of c-fos (+) cells in a coronal section of the anterior OT counterstained with nuclear fast red at P8, P15, P21, P27, and P56. L, lateral; M, medial; D, dorsal; V, ventral. Scale bar: 200 μm.

**Figure 6:**
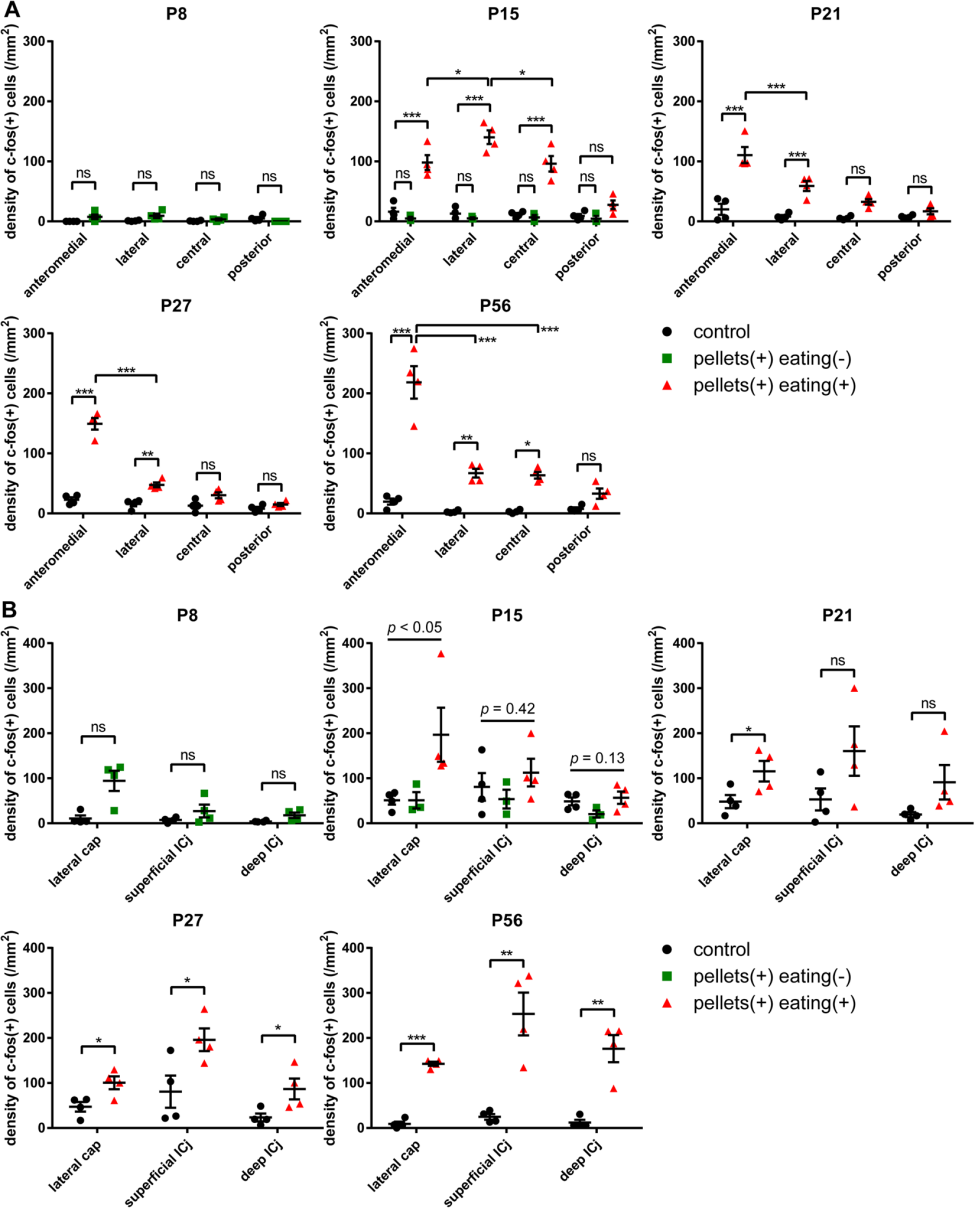
c-fos activation in the individual OT compartments following access and consumption of food pellets in postnatal mice. c-fos mRNA expression after 30 min of food pellet presentation was examined in individual compartments of the OT at P8, P15, P21, P27, and P56. (**A**) Density of c-fos (+) cells in the cortex-like domains. Statistical significance was calculated using two-way ANOVA with *post hoc* Tukey’s test (n = 4). (**B**) Density of c-fos (+) cells in the cap compartment and Islands of Calleja. Statistical significance was calculated using an unpaired *t*-test for P8, P21, P27, and P56, and one-way ANOVA for P15 (n = 4). Black circles, control mice; green squares, mice that were delivered with pellets but did not access or eat; orange triangles, mice that ate delivered pellets. ^*^p < 0.05; ^**^p < 0.01; ^***^p < 0.001; ns, not significant.

As shown in Fig. 1, P8 mice did not eat or approach the food pellets during the test period. c-fos mRNA expression was weak in all of the cortex-like OT domains in P8 mice with or without food pellet presentation (Figs 5 and 6A). In the case of P15 mice presented with food pellets (n = 7), three mice did not eat or approach the food pellets, while the remaining four mice approached and ate the food pellets. c-fos mRNA expression was weak in all of the cortex-like OT domains in the mice that did not approach the food pellets, similar to control mice (P15, green dots; Fig. 6A). By contrast, in the mice that ate the food pellets, c-fos mRNA expression was significantly increased in the OT (P15, red dots; Figs 5 and 6A). The increase in c-fos activation occurred not only in the anteromedial cortex-like domain but also in the lateral and central cortex-like domains. Moreover, the increase in the lateral domain was more prominent compared to that in the anteromedial domain (P15, red dots; Fig. 6A).

All of the P21 mice presented with food pellets approached the food pellets and exhibited eating behaviour, and c-fos mRNA expression significantly increased in the OT. Intriguingly, c-fos activation was more prominent in the anteromedial cortex-like domain compared to the other cortex-like domain (Figs 5 and 6A). While c-fos activation in the anteromedial domain occurred similarly in P15 mice, c-fos activation in the lateral and central domains was attenuated compared to P15 mice. This anteromedial compartment-preferential c-fos activation was further strengthened in P27 and P56 mice presented with food pellets (Figs 5 and 6A). Throughout the examination, c-fos mRNA expression in the OT of control mice (without presentation of food pellets) remained low (Fig. 6A; black dots).

These observations indicated that the OT was consistently activated during food approach and consumption behaviours in postnatal mice. Furthermore, while the c-fos activation occurred in many domains in P15 mice, preferential activation in the anteromedial cortex-like domain occurred in P21 mice. This time period (P21) overlapped with the time period in which mice showed significant food-eating behaviour and became more attracted to food pellets than the lactating mother (see Figs 1 and 2 and Discussion). c-fos mRNA expression in the cap compartments and Islands of Calleja indicated that these cell-dense structures were also activated during the approach and eating behaviour from P15 to P56, whereas structure-specific activation was not evident (Fig. 6B).

### Activation of OT compartments during lactating mother-approach and suckling behaviours in postnatal mice

**Figure 7:**
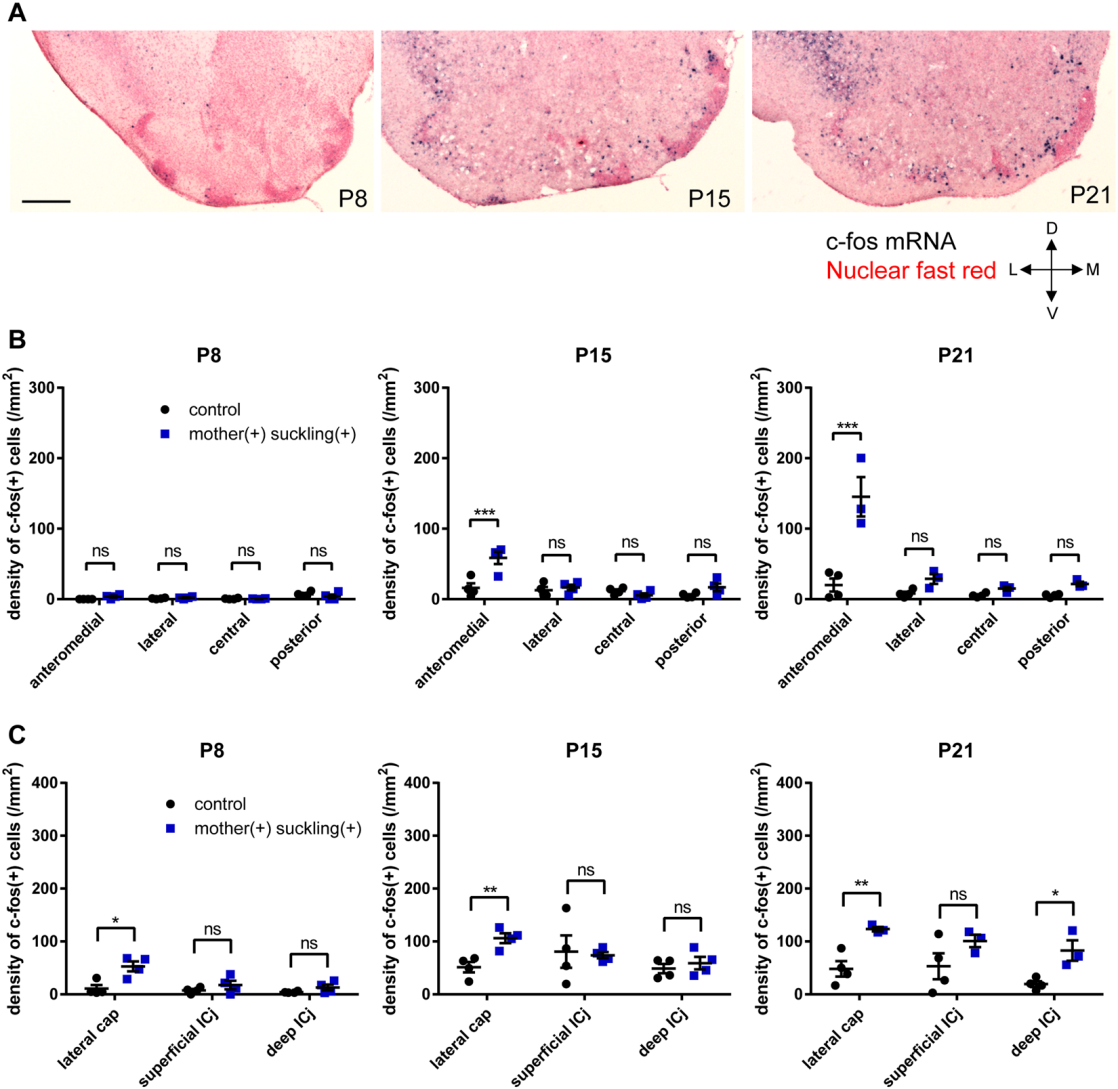
c-fos activation in the OT following access and suckling of lactating mothers in postnatal mice. c-fos mRNA expression after 30 min of lactating mother presentation was examined in individual compartments of the OT at various postnatal periods. (**A**) Distribution of c-fos (+) cells in a coronal section of the anterior OT counterstained with nuclear fast red at P8, P15, and P21. L, lateral; M, medial; D, dorsal; V, ventral. Scale bar: 200 μm. (**B**,**C**) Density of c-fos (+) cells in cortex-like domains (**B**), as well as the cap compartment and Islands of Calleja (**C**). Statistical significance was calculated using two-way ANOVA with *post hoc* Tukey’s test for (**B**) and an unpaired *t*-test for (**C**) (n = 4 for P8 and P15, n = 3 for P21). Black circles, control mice; blue squares, mice suckling mother’s nipple. ^*^p < 0.05; ^**^p < 0.01; ^***^p < 0.001; ns, not significant. Note that the same control group data set used in Fig. 6 was used in Fig. 7.

We further addressed the question of whether the OT was activated during consumption of the mother’s milk in postnatal mice. Mice at P8, P15, and P21 were individually housed in a new cage for 6–16 h without their mothers or food pellets, followed by presentation of lactating mothers. After 30 min of observation, the mice were perfusion-fixed, and c-fos mRNA expression in individual compartments was analysed. Mice not presented with lactating mothers were analysed as controls. As shown in Fig. 1, all P8 mice were attracted to the mothers and attached to the mother’s nipple. However, the OT did not show significant c-fos mRNA expression compared to control mice (Fig. 7A,B). By contrast, in P15 mice attached to the mother’s nipple, the OT showed relatively weak, although significant, c-fos mRNA expression, and activation occurred preferentially in the anteromedial cortex-like domain. Preferential activation of the anteromedial domain was further strengthened in P21 mice attached to the mother’s nipple.

These results indicated that c-fos activation in the OT was not specific to food-pellet eating behaviour but also occurred during access to lactating mothers, and that preferential activation of the anteromedial domain was seen as early as P15 during access to lactating mothers. This suggests that, while the anteromedial OT domain was not involved in the suckling behaviour during the early neonatal period (P8 and before), this domain might have been involved in the suckling behaviour during postnatal maturation (P15 and later). c-fos mRNA expression in the cap compartments and Islands of Calleja suggested that the lateral cap compartment was activated during lactating mother-approach behaviour (Fig. 7).

## Discussion

Here, we examined the shift in feeding behaviour in postnatal mice from mother’s milk-consuming behaviour to environmental food-consuming behaviour, and the structural and functional development of the OT domains during the weaning period. Present results indicated that the acquisition of behaviours for approach to and eating of environmental food occurred concomitantly with the functional development of the OT, particularly the anteromedial domain, during the postnatal period. These results raise the possibility that the anteromedial domain of the OT plays a crucial role in the acquisition of environmental food-eating behaviour during weaning process.

Weaning period is characterised as the developmental period during which suckling and independent feeding co-exist. The present results indicated that the weaning period of mice is similar to that of rats^4–7^. The onset of weaning is defined as the period when pups first begin consuming food other than the mother’s milk, which occurs at approximately P17 in rats^5,6^. Our observations in mice showed similar onset of weaning, where P15 mice began to eat a small amount of food pellets (Fig. 1B). The end of weaning is defined as the period when pups cease to consume the mother’s milk, which occurs at approximately P27 in rats^5,6^. Our observations in mice showed a similar end of weaning, where P27 mice exhibited almost no attraction to lactating mothers (Fig. 1A).

We further examined the attractive behaviour of postnatal mice to lactating mothers and food pellets through individual and simultaneous presentation. Whereas P15 mice primarily depended on the mother’s milk for nutrition and were more attracted to lactating mothers than food pellets, P21 mice showed significant food-eating behaviour, obtained sufficient nutrition from food pellets, and were more attracted to food pellets (Figs 1B,C and 2). Further, the latency to food pellet approach was significantly longer in P15 mice and then shortened in P21 mice as comparable to P27 and P56 mice (Fig. 1A). This transition period from P15 to P21 seems to be the critical time window when the attraction to environmental food is first acquired and strengthened, and then dominates over the attraction to lactating mothers. Thus, the knowledge of structural and functional development of the neuronal circuit for food approach and eating behaviours during this period appears to provide a basis for understanding the neural mechanism underlying the shift in the feeding behaviour during the weaning process.

Present structural and functional analyses have indicated that the OT, particularly the anteromedial domain of the OT, remains immature at birth and develops to maturity during the postnatal period. This is consistent with previous reports showing that ligand binding to D1 and D2 is low at birth and increases significantly within 1 month of birth^28,29^, and that dopamine transporter expression is low at birth and increases postnatally^30^. Neuronal birthdate analyses have indicated that neurons in the medial portion of the OT tend to be born later than those in the lateral portion during the late embryonic period^31^, which might be the basis for the delayed maturation of the anteromedial domain during the postnatal period. Expression of DARPP-32 and D1 in this study suggested that development of the anteromedial OT domain was delayed until P8 and caught up with the lateral domain at P15. Thereafter, the OT at P15, P21, and P56 showed no significant structural differences.

Food approach and eating-induced c-fos mRNA expression pattern in the OT domains differed between P15 and P21. Interestingly, food pellet-induced c-fos activation was observed in all of the OT domains in P15 mice, and the c-fos activation in the lateral domain was greater than that in the anteromedial domain. By contrast, P21 mice showed preferential c-fos activation of the anteromedial domain compared with other domains, which was also the case in adult (P56) mice. This change in food-induced c-fos activation pattern in OT domains occurred concomitantly with the emergence of attractive behaviour to food pellets, raising the possibility that neuronal circuit activity that results in the c-fos induction preferentially in anteromedial OT domain is important in the acquisition of attractive behaviour to environmental food. In adult mice, c-fos induction occurs in the lateral OT domain during aversive behaviour to odour cues^24^. c-fos activation in the lateral domain in P15 mice might represent aversive and fearful responses to unfamiliar food objects at this period. Attenuated c-fos activation in the lateral domain in P21 mice might correspond to the attenuation of aversive and fearful responses, and the increase in the anteromedial domain corresponds to acquisition of attractive responses to food objects. Further examination of lactating mother presentation showed that preferential c-fos activation of the anteromedial domain also occurred during mother’s milk approach and drinking behaviours in P15 mice, indicating that mother’s milk approach and drinking induces c-fos activation preferentially in the anteromedial domain in P15 mice but not in P8 mice. Mother’s milk might activate neural circuit mechanism that results in the preferential c-fos activation in the anteromedial domain in P15 mice, but such neural circuits might be immature in P8 mice. P15 mice might have postnatally acquired neural circuit mechanism involving the anteromedial OT domain to approach mother’s milk in addition to the innate ability to approach mother’s milk.

Fasting of pups by mother deprivation does not significantly influence their milk-consuming behaviour during the initial 2 weeks following birth, whereas fasting begins to enhance milk-consuming behaviour in the third week^6^. This report and our present observations raise the intriguing possibility that the anteromedial OT domain contributes to the fasting-mediated motivation to consume milk. The OT receives inputs from various brain regions including other olfactory cortical areas, the amygdala, and the prefrontal cortex^32–35^ whose functions may be influenced by nutritional state-dependent signals in coordination with the hypothalamus^36–38^. Following maturation at P15, the anteromedial OT might be involved in nutritional state-dependent attractive motivation of feeding.

On the other hand, the OT is unlikely to be involved in milk-consuming behaviour during the early neonatal period until P8, because the OT was structurally immature and was not activated by lactating mother presentation during this period (Figs 3, 4 and 7). While neural circuits responsible for the early attraction to lactating mothers have not yet been identified^39,40^, this notion fits the foregoing supposition that milk-consuming and food-eating behaviours are not unitary processes but independent processes, which are coincidently regulated during weaning^3^. Comparative analyses of neural circuits for lactating mother and environmental food attraction would improve our understanding of how postnatal mice shift feeding habits and become independent from mothers.

The present results do not exclude the possibility that any brain structures other than the OT play a causal role in the weaning process. It should be also noted that the present study did not address the statistical correlation between the behavioural measures and the extent of c-fos activation in OT domains due to the limited number of mice analysed. This study nonetheless offers the basis for further addressing the contribution of the OT domain to the weaning process. Functional manipulation by opto/pharmacogenetics and cell ablation methods which have been successfully adopted also in the adult OT^18–20,23^ would reveal the role of the anteromedial OT domain in the weaning. Weaning is a learning process critical for the development of eating behaviours and for the food preference in adolescence and adult. In adult brain, feeding behaviour recruits various neural circuits that include the hypothalamus, orbitofrontal, insular, medial prefrontal, olfactory, gustatory cortices, and ventral striatum including the anteromedial domain of OT. Further studies of the development of these neural circuits during the weaning period would provide a fundamental knowledge for the acquisition of appropriate feeding behaviours and the development of food preferences.

## Methods

### Animals

All experiments were conducted in accordance with the guidelines of the Physiological Society of Japan and were approved by the Experimental Animal Research Committee of the University of Tokyo and the University of Fukui. C57BL/6 neonatal male mice were purchased from Japan SLC within 1 week after birth with their mothers, housed in plastic cages (W175 × D245 × H125 mm) with paper bedding (Japan SLC Inc., Sizuoka, Japan), and placed in isolation boxes at 26 °C under a 12 h light/dark cycle with the lights turned on at 5:00 A.M. The mice were housed with their mother and littermates until postnatal day 27 (P27), when the mother was removed and the mice were housed in a group of littermates. The mice were fed with food pellets for laboratory animals (MF, Oriental Yeast) and the same food pellets were used in the following behavioural assays.

### Presentation of the feeding object

Prior to presentation of the feeding object and behavioural analyses, mice at different ages (P8, P15, P21, P27, and P56) were individually housed in a plastic cage (W175 × D245 × H125 mm) which was placed in an isolation box. P8, P15, and P21 mice were fasted for 6 h, P27 mice were fasted for 8 h, and P56 mice were fasted for 16 h in the plastic cage. Then, either the mother which was anaesthetised by intraperitoneal sodium pentobarbital injection or one food pellet was delivered in the cage and placed near the P8 mice or the inlet-sided end of the cage (the side near the isolation box door) for the elder mice. The mothers were placed in a supine position, allowing exposure of their nipples. Mouse behaviour at the time of delivery was recorded with a digital video camera and the 30 min period starting from the delivery was analysed (Fig. 1A). In Fig. 2, the mice were individually housed and fasted as described above. Then, the anaesthetised mother and one food pellet were simultaneously delivered near the P8 mice or placed separately at either of the two inlet-sided corners of the cage for the elder mice. In Figs 5–7, the mice were habituated to individual housing and fasting for 1–2 days prior to presentation of the feeding object and behaviour and c-fos mRNA expression analyses. Then the mice were subjected to the same housing, fasting, and food delivery conditions as in Fig. 1A, and anaesthetised for fixation 30 min after food delivery. The control mice in Figs 5–7 were subjected to the same housing and fasting conditions with no food delivery, and anaesthetised for fixation at the same time point as the food-delivered mice.

### Behavioural analyses

The recorded mouse behaviour was visually analysed by the experimenters. In Figs 1A and 5–7, mice that exhibited nipple suckling or the consumption of food pellets even once in the 30 min following delivery were categorised as suckling (+) or eating (+). The duration mice spent sucking nipples or eating food pellets was also measured (Fig. 2).

### Assay of food consumption and body weight with maternal deprivation

In Fig. 1B,C, mice at different ages (P8, P15, and P21) were individually housed in a plastic cage (W175 × D245 × H125 mm). Five to six food pellets and water in a 6 cm petri dish were placed in the cage. The weights of the mice and food pellets were measured every 24 h. The difference in food weight was considered the weight of the diet consumed by the mice (Fig. 1B). The control mice in Fig. 1C were housed with their mother and littermates.

### Sample preparation for histochemistry

Neonatal mice (P3–P8) were anaesthetised on ice. Mice older than P15 were anaesthetised by intraperitoneal injection of sodium pentobarbital and transcardially perfused with phosphate-buffered saline (PBS) followed by 4% paraformaldehyde (PFA). The brain was removed from the skull, immersed in 4% PFA in 0.1 M phosphate buffer (PB) overnight, and transferred to 30% sucrose in RNase-free 0.1 M PB. The brain was embedded in OCT compound (Sakura Finetechnical), frozen at −80 °C, and sliced into coronal sections with a thickness of 25 μm using a cryotome. Sections were rinsed in RNase-free PBS and 0.1 M PB, mounted on slide glasses (CREST; Matsunami Glass Ind., Ltd.) using a paint brush, dried overnight in a vacuum desiccator, and stored at 4 °C until further analyses.

### RNA probe preparation for *in situ* hybridisation

Plasmid templates for *in vitro* transcription of dopamine receptor type 1 (D1) and type 2 (D2) mRNA, and c-fos mRNA, were kindly provided by Dr. Kazuto Kobayashi^41^ and Dr. Hirohide Takebayashi, respectively^42^. We prepared digoxigenin (DIG)-labelled RNA probes for c-fos, D1, and D2 mRNA using an *in vitro* transcription kit (Roche) according to the manufacturer’s instructions.

### *In situ* hybridisation

For Figs 3B, 5 and 7A, *in situ* hybridisation for D1, D2, and c-fos mRNA was performed as previously described with modification^42^. Briefly, coronal sections were fixed in 4% PFA, digested with proteinase K (10 μg/mL) for 30 min, and post-fixed in 4% PFA. Following pre-hybridisation, sections were incubated overnight at 60 °C with DIG-labelled RNA probes. After stringent washing, blocking was performed using 10% normal sheep serum, 1% bovine serum albumin, and 0.1% Triton X-100 in PBS. Then sections were incubated overnight at 4 °C with alkaline phosphatase-conjugated anti-DIG antibody (1:1000 dilution; Roche). Sections were washed in MABT (100 mM maleic acid, 150 mM NaCl, and 0.1% Tween 20) followed by alkaline phosphatase buffer (100 mM NaCl, 100 mM Tris-HCl, pH 9.5, 50 mM MgCl_2_, 0.1% Tween 20, and 5 mM levamisole). Sections were treated with NBT/BCIP (Roche) mixture at room temperature in the dark for colour development. In Figs 5 and 7A, sections were counterstained with nuclear fast red (Vector). Then the sections were dehydrated in ethanol, cleared in Xylene, and mounted in Mount Quick (Daido Sangyo).

### Immunohistochemistry

In Fig. 3B, rabbit anti-DARPP-32 monoclonal antibody (1:400 dilution; Abcam ab40801) and Cy3-conjugated secondary antibody (1:400 dilution; Jackson ImmunoResearch) were used. Sections were incubated with the appropriate antibodies, counterstained with NeuroTrace Green (Thermo Fisher Scientific) and 4′,6-diamidino-2-phenylindole, and mounted in PermaFluor (Thermo Fisher Scientific).

### Reconstruction of domain structures of the OT

We used Neurolucida (MBF Bioscience) to visualise the domain structure of the OT (Fig. 3A), and used the same criteria as a previous report for classification of each domain for all ages analysed^24^ (P3, P8, P15, and P56). Serial coronal sections of the OT (20 μm thickness) from its rostral tip were selected at the rate of one in every two, and the selected sections were analysed. The coronal sections were immunostained with anti-DARPP-32 and anti-NeuN antibodies (data not shown). In each coronal section, we delineated the cap compartment, Islands of Calleja, and cortex-like domains. Cap compartments were identified as a dense cell structure that protruded toward the surface or lateral edge of the OT. Islands of Calleja also represented a dense cell structure located in the superficial layer of the anteromedial OT and deep layer of the central OT. Immunoreactive cap compartments and immunonegative Islands of Calleja were differentiated by DARPP-32 immunostaining. After confirming that the spatial distribution of cap compartments and Islands of Calleja were similar among the different mice, they were identified and discriminated using nuclear fast staining from the cell size, cell density, staining intensity, and spatial localisation, and used as landmarks for OT domain mapping. Cortex-like regions were divided into four domains based on the distribution of cap compartments and Islands of Calleja. If the cortex-like regions were continuous at the gaps of the cap compartments or Islands of Calleja, the boundaries of cortex-like domains were defined by imaginary lines linking the centres of those compartments.

### Image acquisition and quantification

Sections were examined with a bright field microscope (BX51; Olympus, Tokyo, Japan) with the Neurolucida system (MBF Bioscience) (Figs 3B, 5 and 7A) and a fluorescent microscope (BZ-X710; Keyence) (Fig. 3B). To quantify cell density, the area of each domain was delineated and the number of cells was counted using the Neurolucida system. To quantify signal intensity, the area of each domain was delineated and the mean value of the intensity was measured with Photoshop CS6 (Adobe).

### Statistical analyses

Comparisons of latency to food objects (Fig. 1A), body weight (Fig. 1C), time spent in suckling and eating behaviours (Fig. 2B), cell density (Fig. 4A), signal intensity (Fig. 4B–D), and density of c-fos (+) cells (Figs 6 and 7) were performed with GraphPad Prism 6 (Tables 1 and 2). Differences were considered significant at p < 0.05.

**Table 1:** Statistical results for Figs 1, 2 and 4.

Figures	test used	F or t value and degrees of freedom	p value
1A, middle	one-way ANOVA	F (2, 20) = 1.565	P = 0.2337
1A, right	one-way ANOVA	F (3, 33) = 15.15	P < 0.0001
1C, left	two-way ANOVA	F (3, 32) = 0.8029 for ages	P = 0.5015
		F (1, 32) = 61.84 for maternal deprivation	P < 0.0001
		F (3, 32) = 25.55 for interaction	P < 0.0001
1C, middle	two-way ANOVA	F (6, 56) = 43.22 for ages	P < 0.0001
		F (1, 56) = 154.8 for maternal deprivation	P < 0.0001
		F (6, 56) = 3.363 for interaction	P = 0.0067
1C, right	two-way ANOVA	F (6, 56) = 103.1 for ages	P < 0.0001
		F (1, 56) = 3.507 for maternal deprivation	P = 0.0664
		F (6, 56) = 0.2944 for interaction	P = 0.9371
2B, left	one-way ANOVA	F (4, 45) = 39.92	P < 0.0001
2B, right	one-way ANOVA	F (4, 45) = 26.62	P < 0.0001
4A, P3	one-way ANOVA	F (3, 8) = 1.594	P = 0.2654
4A, P8	one-way ANOVA	F (3, 8) = 0.03396	P = 0.9910
4A, P15	one-way ANOVA	F (3, 8) = 0.1996	P = 0.8937
4A, P56	one-way ANOVA	F (3, 8) = 0.6267	P = 0.6177
4B, P3	one-way ANOVA	F (3, 8) = 43.45	P < 0.0001
4B, P8	one-way ANOVA	F (3, 8) = 0.2031	P = 0.8914
4B, P15	one-way ANOVA	F (3, 8) = 1.199	P = 0.3704
4B, P56	one-way ANOVA	F (3, 8) = 0.0769	P = 0.9707
4C, P3	one-way ANOVA	F (3, 8) = 32.36	P < 0.0001
4C, P8	one-way ANOVA	F (3, 8) = 5.36	P = 0.0257
4C, P15	one-way ANOVA	F (3, 8) = 0.5164	P = 0.6825
4C, P56	one-way ANOVA	F (3, 8) = 0.02346	P = 0.9947
4D, P3	one-way ANOVA	F (3, 8) = 0.5496	P = 0.6624
4D, P8	one-way ANOVA	F (3, 8) = 1.474	P = 0.2931
4D, P15	one-way ANOVA	F (3, 8) = 0.4115	P = 0.7493
4D, P56	one-way ANOVA	F (3, 8) = 0.9912	P = 0.4446

**Table 2:** Statistical results for Figs 6 and 7.

Figures	test used	F or t value and degrees of freedom	p value
6A, P8	two-way ANOVA	F (3, 24) = 0.9027 for domains	P = 0.4543
		F (1, 24) = 5.099 for pellets	P = 0.0333
		F (3, 24) = 4.122 for interaction	P = 0.0172
6A, P15	two-way ANOVA	F (3, 32) = 12.62 for domains	P < 0.0001
		F (2, 32) = 149 for pellets, eating	P < 0.0001
		F (6, 32) = 11.63 for interaction	P < 0.0001
6A, P21	two-way ANOVA	F (3, 24) = 22.68 for domains	P < 0.0001
		F (1, 24) = 80.24 for eating	P < 0.0001
		F (3, 24) = 12.64 for interaction	P < 0.0001
6A, P27	two-way ANOVA	F (3, 24) = 87.94 for domains	P < 0.0001
		F (1, 24) = 165 for eating	P < 0.0001
		F (3, 24) = 59.27 for interaction	P < 0.0001
6A, P56	two-way ANOVA	F (3, 24) = 35.49 for domains	P < 0.0001
		F (1, 24) = 131.9 for eating	P < 0.0001
		F (3, 24) = 25.28 for interaction	P < 0.0001
6B, P8 lateral cap	unpaired t-test	t = 3.572 df = 6	P = 0.0118
6B, P8 superficial ICj	unpaired t-test	t = 1.384 df = 6	P = 0.2156
6B, P8 deep ICj	unpaired t-test	t = 2.284 df = 6	P = 0.0625
6B, P15 lateral cap	one-way ANOVA	F (2, 8) = 4.648	P = 0.0458
6B, P15 superficial ICj	one-way ANOVA	F (2, 8) = 0.9599	P = 0.4230
6B, P15 deep ICj	one-way ANOVA	F (2, 8) = 2.646	P = 0.1312
6B, P21 lateral cap	unpaired t-test	t = 2.478 df = 6	P = 0.0479
6B, P21 superficial ICj	unpaired t-test	t = 1.788 df = 6	P = 0.1241
6B, P21 deep ICj	unpaired t-test	t = 1.851 df = 6	P = 0.1137
6B, P27 lateral cap	unpaired t-test	t = 2.954 df = 6	P = 0.0255
6B, P27 superficial ICj	unpaired t-test	t = 2.633 df = 6	P = 0.0389
6B, P27 deep ICj	unpaired t-test	t = 2.537 df = 6	P = 0.0443
6B, P56 lateral cap	unpaired t-test	t = 19.81 df = 6	P < 0.0001
6B, P56 superficial ICj	unpaired t-test	t = 4.77 df = 6	P = 0.0031
6B, P56 deep ICj	unpaired t-test	t = 5.324 df = 6	P = 0.0018
7B, P8	two-way ANOVA	F (3, 24) = 6.108 for domains	P = 0.0031
		F (1, 24) = 0.164 for suckling	P = 0.6891
		F (3, 24) = 2.647 for interaction	P = 0.0720
7B, P15	two-way ANOVA	F (3, 24) = 13.56 for domains	P < 0.0001
		F (1, 24) = 13.25 for suckling	P = 0.0013
		F (3, 24) = 8.65 for interaction	P = 0.0005
7B, P21	two-way ANOVA	F (3, 20) = 25.99 for domains	P < 0.0001
		F (1, 20) = 40.41 for suckling	P < 0.0001
		F (3, 20) = 16.55 for interaction	P < 0.0001
7C, P8 lateral cap	unpaired t-test	t = 3.679 df = 6	P = 0.0103
7C, P8 superficial ICj	unpaired t-test	t = 1.203 df = 6	P = 0.2742
7C, P8 deep ICj	unpaired t-test	t = 1.613 df = 6	P = 0.1578
7C, P15 lateral cap	unpaired t-test	t = 4.247 df = 5	P = 0.0081
7C, P15 superficial ICj	unpaired t-test	t = 1.564 df = 5	P = 0.1787
7C, P15 deep ICj	unpaired t-test	t = 3.63 df = 5	P = 0.0151
7C, P21 lateral cap	unpaired t-test	t = 4.07 df = 6	P = 0.0066
7C, P21 superficial ICj	unpaired t-test	t = 0.2187 df = 6	P = 0.8341
7C, P21 deep ICj	unpaired t-test	t = 0.6948 df = 6	P = 0.5132

## Data Availability

In publication we make materials, data and associated protocols promptly available to readers without undue qualifications in material transfer agreements.

## References

[1] Henning SJ (1981). Postnatal development: coordination of feeding, digestion, and metabolism. Am. J. Physiol..

[2] Ost’adalova I, Babicky A (2012). Periodization of the early postnatal development in the rat with particular attention to the weaning period. Physiol. Res..

[3] Thiels E, Alberts JR, Cramer CP (1990). Weaning in rats: II. Pup behavior patterns. Dev. Psychobiol..

[4] Babicky A, Ostadalova I, Parizek J, Kolar J, Bibr B (1970). Use of radioisotope techniques for determining the weaning period in experimental animals. Physiol. Bohemoslov..

[5] Babicky A, Parizek J, Ostadalova I, Kolar J (1973). Initial solid food intake and growth of young rats in nests of different sizes. Physiol. Bohemoslov..

[6] Henning SJ, Chang SS, Gisel EG (1979). Ontogeny of feeding controls in suckling and weanling rats. Am. J. Physiol..

[7] Thiels E, Alberts JR (1985). Milk availability modulates weaning in the Norway rat (Rattus norvegicus). J. Comp. Psychol..

[8] Birch LL (1999). Development of food preferences. Annu. Rev. Nutr..

[9] Oliveira-Maia, A. J., Simon, S. A. & Nicolelis, M. A. L. In *Methods for Neural Ensemble Recordings* (eds nd & Nicolelis, M. A. L.) (CRC Press/Taylor & Francis Taylor & Francis Group, LLC., 2008).

[10] Berridge KC, Kringelbach ML (2015). Pleasure systems in the brain. Neuron.

[11] Wesson DW, Wilson DA (2011). Sniffing out the contributions of the olfactory tubercle to the sense of smell: hedonics, sensory integration, and more?. Neurosci. Biobehav. Rev..

[12] Xiong A, Wesson DW (2016). Illustrated review of the ventral striatum’s olfactory tubercle. Chemical Senses.

[13] Yamaguchi M (2017). Functional Sub-circuits of the olfactory system viewed from the olfactory bulb and the olfactory tubercle. Front. Neuroanat..

[14] Luskin MB, Price JL (1983). The laminar distribution of intracortical fibers originating in the olfactory cortex of the rat. J. Comp. Neurol..

[15] Ikemoto S (2007). Dopamine reward circuitry: two projection systems from the ventral midbrain to the nucleus accumbens-olfactory tubercle complex. Brain Res. Rev..

[16] Millhouse OE, Heimer L (1984). Cell configurations in the olfactory tubercle of the rat. J. Comp. Neurol..

[17] Narikiyo K, Manabe H, Mori K (2014). Sharp wave-associated synchronized inputs from the piriform cortex activate olfactory tubercle neurons during slow-wave sleep. J. Neurophysiol..

[18] Agustin-Pavon C, Martinez-Garcia F, Lanuza E (2014). Focal lesions within the ventral striato-pallidum abolish attraction for male chemosignals in female mice. Behav. Brain Res..

[19] DiBenedictis BT, Olugbemi AO, Baum MJ, Cherry JA (2015). DREADD-induced silencing of the medial olfactory tubercle disrupts the preference of female mice for opposite-sex chemosignals(1,2,3). eNeuro.

[20] Fitzgerald BJ, Richardson K, Wesson DW (2014). Olfactory tubercle stimulation alters odor preference behavior and recruits forebrain reward and motivational centers. Front. Behav. Neurosci..

[21] Gadziola MA, Tylicki KA, Christian DL, Wesson DW (2015). The olfactory tubercle encodes odor valence in behaving mice. J Neurosci.

[22] Gadziola MA, Wesson DW (2016). The neural representation of goal-directed actions and outcomes in the ventral striatum’s olfactory tubercle. J. Neurosci..

[23] Zhang Z (2017). Activation of the dopaminergic pathway from VTA to the medial olfactory tubercle generates odor-preference and reward. eLife.

[24] Murata K, Kanno M, Ieki N, Mori K, Yamaguchi M (2015). Mapping of learned odor-induced motivated behaviors in the mouse olfactory tubercle. J. Neurosci..

[25] Fallon JH, Riley JN, Sipe JC, Moore RY (1978). The islands of Calleja: organization and connections. J. Comp. Neurol..

[26] Hosoya Y, Hirata Y (1974). The fine structure of the “dwarf-cell cap” of the olfactory tubercle in the rat’s brain. Arch. Histol. Jpn..

[27] Ouimet CC, Miller PE, Hemmings HC, Walaas SI, Greengard P (1984). DARPP-32, a dopamine- and adenosine 3′:5′-monophosphate-regulated phosphoprotein enriched in dopamine-innervated brain regions. III. Immunocytochemical localization. J. Neurosci..

[28] Schambra UB (1994). Ontogeny of D1A and D2 dopamine receptor subtypes in rat brain using *in situ* hybridization and receptor binding. Neuroscience.

[29] Tarazi FI, Tomasini EC, Baldessarini RJ (1999). Postnatal development of dopamine D1-like receptors in rat cortical and striatolimbic brain regions: An autoradiographic study. Dev. Neurosci..

[30] Coulter CL, Happe HK, Murrin LC (1996). Postnatal development of the dopamine transporter: a quantitative autoradiographic study. Brain Res. Dev. Brain Res..

[31] Bayer SA (1985). Neurogenesis in the olfactory tubercle and islands of Calleja in the rat. Int. J. Dev. Neurosci..

[32] Berendse HW, Galis-de Graaf Y, Groenewegen HJ (1992). Topographical organization and relationship with ventral striatal compartments of prefrontal corticostriatal projections in the rat. J. Comp. Neurol..

[33] Gutierrez-Castellanos N, Pardo-Bellver C, Martinez-Garcia F, Lanuza E (2014). The vomeronasal cortex - afferent and efferent projections of the posteromedial cortical nucleus of the amygdala in mice. Eur. J. Neurosci..

[34] Haberly LB, Price JL (1978). Association and commissural fiber systems of the olfactory cortex of the rat. J. Comp. Neurol..

[35] Neville, K. R. & Haberly, L. B. In *The s*ynaptic *o*rgani*zation of the brain, 5th edition* (ed G.M. Shepherd) 415–454 (Oxford University Press, 2004).

[36] Chen Y, Knight ZA (2016). Making sense of the sensory regulation of hunger neurons. Bioessays.

[37] Julliard AK, Al Koborssy D, Fadool DA, Palouzier-Paulignan B (2017). Nutrient sensing: another chemosensitivity of the olfactory system. Front. Physiol..

[38] Rossi MA, Stuber GD (2018). Overlapping brain circuits for homeostatic and hedonic feeding. Cell Metab..

[39] Logan DW (2012). Learned recognition of maternal signature odors mediates the first suckling episode in mice. Curr. Biol..

[40] Perry RE, Blair C, Sullivan RM (2017). Neurobiology of infant attachment: attachment despite adversity and parental programming of emotionality. Curr. Opin. Psychol..

[41] Sano H (2003). Conditional ablation of striatal neuronal types containing dopamine D2 receptor disturbs coordination of basal ganglia function. J. Neurosci..

[42] Bepari AK, Watanabe K, Yamaguchi M, Tamamaki N, Takebayashi H (2012). Visualization of odor-induced neuronal activity by immediate early gene expression. BMC neuroscience.

